# Transcatheter Versus Sutureless Aortic Valve Replacement: A Propensity-Matched Single-Center Cohort Study

**DOI:** 10.3390/medicina62030476

**Published:** 2026-03-03

**Authors:** Nikoleta Stanitsa, Emmanouel Tempelis, Ilias Samiotis, Dimitris Oikonomou, Konstantinos Triantafyllou, George Lazopoulos, Periklis Tomos, Panagiotis Dedeilias

**Affiliations:** 1 Cardiac Surgery Department, Evangelismos General Hospital, 106 76 Athens, Greecesamiotisilias@gmail.com (I.S.); pdedeilias@gmail.com (P.D.); 2 Cardiology Department, Evangelismos General Hospital, 106 76 Athens, Greece; 3 Cardiac Surgery Department, University Hospital, 715 00 Heraklion, Greece; lazopoulosg@ath.forthnet.gr; 4 Thoracic Surgery Department, Attikon University Hospital, 12 462 Athens, Greece

**Keywords:** aortic valve replacement, sutureless valve, Perceval, transcatheter aortic valve implantation

## Abstract

*Background and Objectives*: Over the past decade, transcatheter aortic valve replacement (TAVI) has evolved from a treatment for inoperable patients to an established option across all risk categories. In parallel, the Perceval sutureless valve has demonstrated safety and efficacy especially for minimally invasive surgical aortic valve replacement (AVR). Despite the advances of both TAVI and Perceval, robust long-term data and clear patient selection criteria are still lacking. This retrospective single-center study reports the outcomes of patients undergoing isolated AVR with the Perceval sutureless valve or with TAVI. *Materials and Methods*: We retrospectively reviewed consecutive patients undergoing isolated AVR at our institution between April 2013 and December 2024. Of 1006 eligible patients (424 TAVI; 582 Perceval), propensity score matching was performed for age, sex, EuroSCORE II, body surface area, and comorbidities, yielding 197 matched pairs. Primary endpoints were all-cause and cardiovascular mortality. Secondary endpoints included acute kidney injury, permanent pacemaker implantation, stroke, pericardial effusion, ICU stay, and overall hospital stay. Clinical and echocardiographic follow-up was obtained by medical-record review and routine echocardiography, with an additional prospective clinical and echocardiographic evaluation at 6–12 months. *Results*: Postprocedural paravalvular leak was significantly more frequent after TAVI than after Perceval AVR (23.4% vs. 2.5%; *p* < 0.001). At 6–12 months, TAVI was associated with greater aortic regurgitation and higher rates of para- and intra-prosthetic leak (both *p* < 0.001) and higher mean transvalvular gradients, particularly in small and medium valve sizes. ICU and overall hospital stay were longer after Perceval implantation (both *p* < 0.001). New permanent pacemaker implantation was numerically higher after TAVI (11.2% vs. 5.6%; *p* = 0.063). Early mortality was similar; however, 1-year mortality was higher after TAVI (16.2% vs. 9.1%; *p* = 0.045), and Kaplan–Meier analysis demonstrated better overall survival with Perceval (*p* < 0.001), while cardiovascular survival did not differ significantly (*p* = 0.851). *Conclusions*: Our study underscores the importance of meticulous patient selection when choosing between TAVI and Perceval. Perceval implantation was associated with better long-term overall survival than TAVI in the propensity-matched cohort. Paravalvular leaks were more frequent after TAVI and associated with poorer survival. Both approaches achieve excellent outcomes; however, differences in long-term survival and valve performance highlight the need for a personalized treatment strategy guided by a multidisciplinary heart team.

## 1. Introduction

TAVI was originally developed for elderly patients with severe aortic stenosis deemed high risk for surgery and has since achieved broad clinical uptake [1]. Over the years, its use evolved into an established option across all risk categories [2,3], with the recent European guidelines recommending TAVI in patients ≥ 70 years of age with tricuspid aortic valve stenosis, if the anatomy is suitable [4]. In parallel, surgical AVR has evolved through the introduction of sutureless and rapid-deployment valves designed to reduce procedural invasiveness [5]. The Perceval valve (Corcym SRL, Saluggia, Italy) is one such sutureless device, featuring a self-expanding nitinol frame with shape memory that enables automatic deployment and positioning [6,7].

Despite the advances of both TAVI and Perceval use, current guidelines do not provide specific recommendations regarding their use, because no randomized controlled trials directly compare TAVI with sutureless or rapid-deployment valves and robust long-term data are missing [4]. Existing evidence is largely based on retrospective analyses, and important differences remain since TAVI is typically offered to older patients with higher operative risk [8,9,10,11,12]. In contemporary practice, the choice between transcatheter AVR and surgical AVR is individualized and driven by multidisciplinary heart team assessment. This study was designed to inform decision making in patients with severe aortic valve disease referred for isolated AVR in a real-world setting, including the “intermediate” clinical space in which both transcatheter and surgical strategies may be technically feasible, but selection is influenced by factors that are not always fully captured in retrospective datasets (e.g., granular frailty metrics, detailed anatomical complexity, vascular access constraints, and patient preference). Accordingly, our findings should be interpreted as comparative strategy outcomes within institutional heart team practice rather than as evidence of complete treatment interchangeability.

The aim of this propensity-score-matched study was to compare outcomes of sutureless AVR and TAVI in a cohort with balanced baseline clinical characteristics, acknowledging the inherent limitations of observational matching.

## 2. Materials and Methods

All patients with severe aortic valve disease undergoing isolated aortic valve intervention at our institution between April 2013 and December 2024 were screened for inclusion. Exclusion criteria were the presence of concomitant cardiac surgery, active endocarditis, prior aortic surgery, emergency operation and incomplete follow-up.

This retrospective study was approved by the local Ethics Committee (Scientific Congress of Evangelismos Hospital, Protocol Number 237). Informed consent was waived given the retrospective nature of this study.

To minimize baseline imbalances between groups, we performed propensity score matching based on age, sex, EuroSCORE II, body surface area, and comorbidities (i.e., chronic pulmonary disease, coronary artery disease, AF, diabetes mellitus, dialysis, hypertension and dyslipidemia). The matching covariates were selected because they were consistently available across the entire study period and represent major baseline clinical factors known to influence both treatment selection and outcomes.

After matching, 197 patients were included in each group. Matching was done using a 1:1 nearest-neighbor matching method without replacement. A fixed caliper width (match tolerance) of 0.06 was applied. This specific width was determined iteratively to achieve optimal balance between the groups while maximizing the retained sample size. Following the matching procedure, the balance was assessed using standardized mean differences (SMDs). All covariates achieved an SMD < 0.10, indicating that the groups were well-balanced and comparable for subsequent analysis (Austin, 2011 [13]).

Treatment allocation (TAVI vs. sutureless surgical AVR with the Perceval prosthesis) was determined by a multidisciplinary heart team (cardiac surgeons, interventional and non-interventional cardiologists, imaging specialists, and anesthesiologists). Decisions were individualized based on an integrated assessment of: (i) operative risk (EuroSCORE II and clinical judgement), (ii) anatomical suitability for each approach (including annular/root anatomy and feasibility of valve sizing), (iii) vascular access feasibility and anticipated access-related risk for transfemoral TAVI, (iv) frailty/functional status and comorbidity burden, and (v) patient preference after counseling. In routine institutional practice, Perceval sutureless AVR was selected when a surgical approach was favored but procedural simplification and/or minimally invasive facilitation was desirable, whereas TAVI was preferred when a less invasive transcatheter strategy was favored and anatomical and vascular criteria were met.

In this context, “eligible for both” refers to patients in whom either strategy was technically feasible and clinically acceptable according to heart team assessment; however, the final decision remained individualized. Propensity score matching was used to reduce baseline differences between groups but cannot fully account for unmeasured confounders inherent to observational studies.

The primary endpoints were cardiovascular and all-cause mortality. Secondary endpoints included acute kidney injury (AKI), permanent pacemaker implantation (PPI), stroke, pericardial effusion, intensive care unit (ICU) stay, and overall hospital stay. Clinical and echocardiographic follow-up was conducted retrospectively through review of electronic medical records and routine echocardiographic examinations, with an additional prospective clinical and echocardiographic evaluation performed at 6–12 months after surgery.

Paravalvular and intra-prosthetic regurgitation were assessed using transthoracic echocardiography in accordance with contemporaneous guideline recommendations at the time of examination. Severity grading was based on an integrated qualitative and semi-quantitative approach, including color Doppler jet extent and hemodynamic impact, as documented in institutional echocardiography reports. All echocardiographic studies were performed and interpreted by experienced cardiologists specialized in cardiac imaging. Given the retrospective nature of the study, echocardiographers were not formally blinded to the implanted valve type.

### 2.1. Study Device

The Perceval valve is a sutureless surgical aortic bioprosthesis indicated for AVR. The device consists of a functional component made of bovine pericardial tissue, treated and stabilized in a buffered glutaraldehyde solution, mounted on a self-expanding super-elastic nitinol stent. The prosthesis is supplied in an aldehyde-free storage solution. The Perceval Plus variant incorporates the FREE^®^ tissue treatment, an advanced anti-calcification technology [14,15]. Before implantation, the valve is collapsed to an appropriate diameter and loaded onto a dedicated delivery system. It is positioned under direct visualization within the aortic annulus and released into place, after which postdilatation is performed using a dedicated balloon catheter to ensure complete expansion and optimal seating.

TAVI prostheses are catheter-delivered aortic bioprostheses indicated for the treatment of severe native aortic valve disease and, when applicable, failed surgical bioprosthetic valves (valve-in-valve). The device consists of a functional valve component made of bovine or porcine pericardial tissue mounted on a metallic stent frame and delivered in a crimped state via a dedicated delivery system, most commonly through transfemoral access. Under fluoroscopic guidance (with echocardiographic assessment as needed), the prosthesis is positioned across the native annulus and deployed to anchor within the aortic root. Two principal mechanisms of expansion were used: self-expanding valves (Evolut R/PRO, Medtronic plc, Galway, Ireland; ACURATE/ACURATE neo, Boston Scientific Corporation, Marlborough, MA, USA; Allegra, NVT GmbH (New Valve Technology), Hechingen, Germany; Portico/Navitor, Abbott, Abbott Park, IL, USA; Hydra, Vascular Innovations Co., Ltd., Nonthaburi, Thailand) and balloon-expandable valves (SAPIEN 3, Edwards Lifesciences Corporation, Irvine, California, USA; Myval, Meril Life Sciences Pvt. Ltd., Vapi, Gujarat, India). The deployment occurs either by gradual release of a nitinol frame (self-expanding) or by balloon inflation to achieve immediate fixation. After deployment, valve position and function are confirmed angiographically and/or echocardiographically, and postdilatation may be performed when indicated to optimize expansion and reduce residual paravalvular regurgitation.

### 2.2. Statistical Analysis

Quantitative variables were expressed as mean values and standard deviation or as median and interquartile range (IQR), while categorical variables were expressed as absolute and relative frequencies. McNemar and paired samples t-tests or Wilcoxon signed-rank tests were used for the comparison of qualitative and quantitative variables between the two groups. A Mann–Whitney test was used for the comparison of mean pressure gradient (MPG) between the two procedures within each valve size. Kaplan–Meier survival estimates for overall and cardiovascular disease (CVD) survival were graphed over the follow-up period. A log-rank test was used for the comparison of survival curves between TAVI and Perceval cases. Life table analyses were used to calculate cumulative survival rate (standard errors) for specific time intervals. The prognostic value of having any degree of paravalvular leak, any degree of intra-prosthetic leak and aortic regurgitation (AR) > 2 was assessed by Cox regression analysis. Multivariate Cox proportional hazard models were used to determine the independent predictors for overall and CVD-related death. The assumption of proportional hazards was evaluated by testing for interaction with a continuous time variable. Hazard ratios (HRs) with 95% confidence intervals (95% CI) were computed from the Cox regression analyses. To account for the paired nature of the matched cohort, Cox proportional hazard models with a robust (sandwich) variance estimator were used; additionally, a Fine–Gray competing-risk model was applied for cardiovascular mortality. Type of procedure, gender, age, EuroSCORE II, comorbidities, paravalvular and intra-prosthetic leaks, aortic regurgitation > 2, PPM implant, pericardial effusion, AKI, stroke and mean pressure gradient were entered as covariates. All two-way interaction terms of type of procedure with the rest of the covariates were tested and reported if significant. No imputation for missing data was performed. All reported *p* values were two-tailed. Statistical significance was set at *p* < 0.05 and analyses were conducted using SPSS statistical software (version 27.0). No formal adjustment for multiple comparisons was applied across secondary endpoints; these analyses should be interpreted as exploratory.

Reporting of this observational study follows the STROBE guidelines; the STROBE checklist is provided as Supplementary Material.

## 3. Results

Data from 1006 patients were collected (424 TAVI and 582 Perceval cases). After the propensity score matching, the final sample consisted of 394 patients (197 TAVI and 197 Perceval. Participants’ demographical characteristics as well as data from their medical history are presented in Table 1, before and after the propensity score matching. After matching, baseline characteristics were well-balanced (all SMDs < 0.10; Table 1). Former or current smokers were 26.9% (*n* = 53) of the TAVI group and 33.5% (*n* = 66) of the Perceval group (*p* = 0.218). Preoperative ultrasound results are provided in Table S1 of the Supplementary Material. Preoperative LVEF did not differ between groups (*p* > 0.05). Patients’ immediate postoperative echocardiographic results are presented by group in Table 2. The rate of any paravalvular leak was significantly greater in the TAVI group (23.4% vs. 2.5%; *p* < 0.001). Postoperative complications by group are presented in Table 3. Major vascular complications requiring intervention occurred in 4.6% of TAVI patients; minor events occurred in 4.1% and were treated conservatively. Patients who underwent Perceval AVR had significantly greater overall and ICU stay (*p* < 0.001 for both comparisons). Echocardiographic data at 6–12 months after surgery are presented by group in Table S2 of the Supplementary Material. Aortic regurgitation was significantly more severe in the TAVI group (*p* < 0.001). Also, the intra-prosthetic and paravalvular leak percentages were significantly greater in TAVI cases (*p* < 0.001 for both comparisons). In addition, MPG was significantly higher in TAVI cases (*p* < 0.001) while LVEF was similar in both groups (*p* > 0.05). LVEF was similar at 6–12 months compared to the preoperative values (*p* > 0.05) in the TAVI cases, while it increased significantly (*p* < 0.001) in the Perceval cases. MPG diminished significantly at 6–12 months compared to the preoperative ones in both groups (*p* < 0.001). MPGs were compared between the two procedures stratified by valve size and it was observed that, in small (*p* < 0.001) (nominal diameter 20–23 mm) and medium valve sizes (*p* < 0.001) (nominal diameter 24–26 mm), MPG was significantly greater in the TAVI group, while in the large valve size (nominal diameter ≥ 27 mm) there was no significant difference between the two groups (*p* = 0.872) (Table 4).

**Figure 1:**
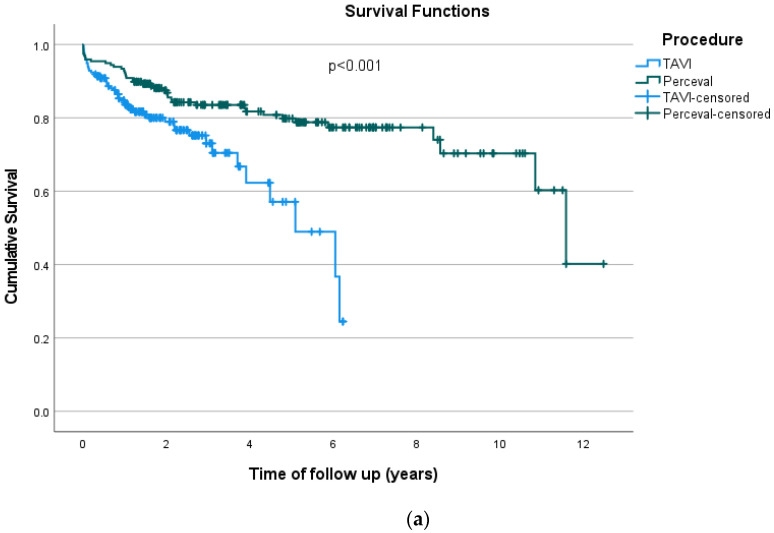

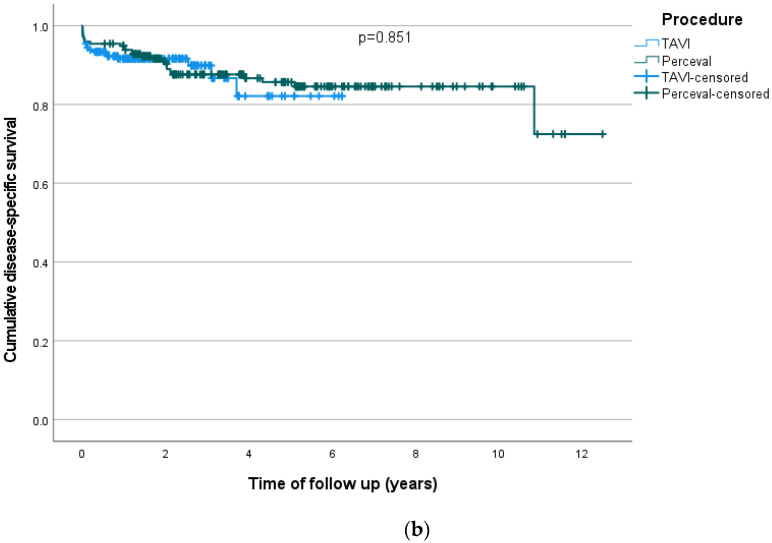
Kaplan–Meier survival curves for overall survival (**a**) and for disease-specific survival (**b**) by procedure.

The percentage of periprocedural death was 0.5% in both procedures and the in-hospital death was 3.0% in the TAVI group and 4.1% in the Perceval group. In the TAVI group the 30-day mortality was 6.1%, 9.1% at 6 months, 16.2% at 1 year and 21.8% at 3 years. Corresponding figures for the Perceval group were 4.6% for 30-day, 6.6% for 6-month, 9.1% for 1-year and 15.7% for 3-year mortality. Mean survival time for the overall population was 8.67 years (SE = 0.37 years), 4.34 years (SE = 0.24 years) for TAVI and 9.50 years (SE = 0.41 years) for Perceval. Kaplan–Meier curves for overall survival are presented in Figure 1a, by procedure. The overall survival was significantly greater in the Perceval group (*p* < 0.001). The percentage of cardiovascular death was 9.6% in the TAVI group and 13.2% in the Perceval group; *p* > 0.05. Mean disease-specific survival time for the total sample was 10.58 years (SE = 0.31 years). For the TAVI group, mean disease-specific survival time was 5.44 years (SE = 0.20 years) and for the Perceval group it was 10.62 years (SD = 0.354 years). Kaplan–Meier curves for CVD death are presented in Figure 1b for each procedure; they were not significantly different (*p* = 0.851). Adjusting for competing risks, the difference remained non-significant (HR = 0.88; 95% CI: 0.47–1.66; *p* = 0.692). Life table analysis revealed that, in TAVI cases, the probability of overall survival at 1 year was 83.8% (SE = 2.7%), 72.8% at 3 years (SE = 4.0%) and 58.2% at 5 years (SE = 7.7%). In Perceval cases, the probability of overall survival at 1 year was 92.4% (SE = 1.9%), 83.1% at 3 years (SE = 2.8%) and 79.5% at 5 years (SE = 3.2%). Regarding disease-specific survival it was found that, in TAVI cases, the probability of survival at 1 year was 91.0% (SE = 2.1%), 89.3% at 3 years (SE = 2.7%) and 81.7% at 5 years (SE = 5.7%). In Perceval cases, the probability of disease-specific survival at 1 year was 94.9% (SE = 1.6%), 87.1% at 3 years (SE = 2.6%) and 85.2% at 5 years (SE = 2.9%). Having any paravalvular leak, any intra-prosthetic leak and AR > 2 were significantly associated with greater overall hazard as well as hazard for CVD death in both procedures; *p* < 0.05 (Table 5). The effect of having any paravalvular leak was significantly greater in Perceval patients concerning both overall survival (HR of interaction term = 13.51; 95% CI: 4.29–42.54; *p* < 0.001) and survival of CVD (HR of interaction term = 11.53; 95% CI: 2.92–45.55; *p* < 0.001) compared to patients who underwent TAVI. Also, the effect of having AR > 2 on overall survival was significantly greater in Perceval patients (HR of interaction term = 14.12; 95% CI: 2.34–88.94; *p* = 0.004).

After multiple regression via Cox models, it was found that cases with AR > 2 had 19.65 times greater overall hazard (HR = 19.65; 95% CI: 5.85–65.97; *p* < 0.001) and 24 times greater hazard for CVD death (HR = 24; 95% CI: 5.8–99.23; *p* < 0.001) (Table 6). Also, cases who had had a stroke had significantly greater overall hazard (HR = 3.90; 95% CI: 1.74–8.74; *p* = 0.001). When two-way interaction terms of type of procedure with the rest of the covariates were entered in the analysis, it was found that only the interaction of paravalvular leaks with type of procedure was significant for overall survival (HR = 13.34; 95% CI: 3.60–49.41; *p* < 0.001) and CVD death (HR = 10.00; 95% CI: 2.12–47.09; *p* = 0.004), with the main effect of paravalvular leaks remaining non-significant, indicating that only patients with paravalvular leaks who had undergone the Perceval procedure had significantly greater hazard. The rest of the interaction terms were not found to be significant (*p* > 0.05).

## 4. Discussion

In our matched cohort, TAVI was associated with a markedly higher burden of residual regurgitation and less favorable follow-up hemodynamics than Perceval sutureless AVR, while early major complications were broadly comparable. Kaplan–Meier analysis showed significantly better overall survival with Perceval, whereas cardiovascular mortality did not differ, and this remained non-significant after competing-risk adjustment using Fine–Gray regression. This divergence suggests that differences in non-cardiovascular death may contribute to the overall survival separation and is consistent with the broader concern that, in observational cohorts, treatment allocation may be influenced by unmeasured vulnerability (e.g., frailty or non-cardiac comorbidity burden) even after propensity matching; contemporary meta-analytic comparisons similarly emphasize sensitivity of cause-specific outcomes to baseline risk differences and competing risks during long follow-up [10].

The most consistent differentiator was paravalvular leak after the procedure, which was significantly more frequent after TAVI (23.4% vs. 2.5%, *p* < 0.001). This finding aligns closely with the multicenter comparative experience reported by Biancari et al., where TAVI demonstrated substantially higher rates of both mild and moderate–severe paravalvular regurgitation compared with Perceval AVR, supporting the direction and clinical relevance of our early echocardiographic results [8].

Importantly, the excess regurgitation in our TAVI cohort persisted beyond discharge: at 6–12 months, TAVI was associated with more severe aortic leak and markedly higher rates of paraprosthetic leak (26.5% vs. 1.5%) and intra-prosthetic leak (8.2% vs. 0.5%). A similar PVL signal has been reported in the device-specific comparison by Gerfer et al. (Perceval-S vs. ACURATE neo/TF), in which PVL was essentially absent after Perceval-S but frequent after TAVI, supporting the reproducibility of this echocardiographic pattern across platforms [9].

Beyond being a procedural descriptor, regurgitation appeared prognostically relevant in our dataset: AR > 2 (and leak variables) was strongly associated with overall and cardiovascular hazards and remained a dominant independent predictor in multivariable models in both treatment groups. This supports a plausible mechanistic pathway by which the higher postprocedural regurgitation burden after TAVI may contribute to adverse mid-term outcomes; however, very large hazard ratios can occur when events are rare within matched samples and should be interpreted cautiously as potentially unstable effect-size estimates, with emphasis placed on the direction and consistency of association rather than precise magnitude [10].

At follow-up (6–12 months), mean gradients were higher after TAVI (13.77 vs. 10.33 mmHg, *p* < 0.001), despite comparable baseline hemodynamics. The gradient disadvantage was most pronounced in small and medium valve sizes, while gradients were similar in large valves, suggesting that annular size modifies comparative hemodynamic performance in routine practice. This adds an important nuance to the comparative literature by indicating that, in smaller annuli, the combined burden of residual regurgitation and higher gradients after TAVI may be particularly clinically relevant and supports integrating gradients and PVL rather than interpreting either metric in isolation [10].

In our study, new permanent pacemaker implantation was numerically higher after TAVI (11.2% vs. 5.6%) but did not reach statistical significance (*p* = 0.063). Our two-year endpoint captured meaningful differences in timing—pacing occurred early after TAVI (median 2 days) but later after Perceval (median 190 days)—highlighting how endpoint definition windows and longer follow-up may attenuate early between-strategy differences. This sits between published comparative observations and is consistent with the conclusion of the recent systematic review/meta-analysis by Ali-Hasan-Al-Saegh et al., which did not demonstrate a statistically significant difference in pacemaker implantation between Perceval AVR and TAVI across available studies [10].

As expected for an operative strategy, ICU and total hospital length of stay were longer after Perceval AVR (both *p* < 0.001). This “trade-off profile” is consistent with the broader synthesis of comparative evidence describing a profile exchange between strategies—surgical recovery and perioperative bleeding/atrial fibrillation risks on the one hand versus access-related complications and valve–annulus interaction risks (notably PVL) on the other—reinforcing the importance of individualized heart team decision making that integrates baseline risk, anatomical suitability, annular size, access feasibility, and the downstream clinical consequences of residual regurgitation and gradients [10].

Although early mortality was similar, 1-year mortality was higher after TAVI (16.2% vs. 9.1%, *p* = 0.045), and overall survival was significantly better after Perceval, while cardiovascular mortality curves were not significantly different. These findings are concordant with the “grey-zone” comparative study by Muneretto et al., where self-expanding TAVI was associated with more perioperative complications (including pacemaker implantation) and inferior 24-month event-free survival compared with surgery and sutureless AVR, supporting the clinical relevance of careful strategy selection in patients who may be technically eligible for either approach [11].

In the fully adjusted Cox model (Table 6), the treatment strategy (Perceval vs. TAVI) was not independently associated with overall survival (HR 0.63, *p* = 0.115) or cardiovascular death (HR 1.89, *p* = 0.149), suggesting that long-term risk in this matched cohort is primarily driven by postprocedural events and valve-related sequelae rather than procedural assignment alone. This is compatible with contemporary perspectives that comparative strategy outcomes are heavily influenced by residual valve dysfunction and downstream complications, particularly when baseline risk differences are minimized [16].

The strongest predictor in Table 6 was AR > 2, which was associated with markedly increased risk for both overall mortality (HR 19.65, *p* < 0.001) and cardiovascular death (HR 24.0, *p* < 0.001). The prognostic importance of post-TAVI regurgitation has been repeatedly demonstrated, including large analyses showing that even residual AR/PVL after transcatheter implantation is associated with adverse longer-term outcomes, supporting a plausible pathway linking incomplete sealing/residual leak to late hazard [16].

In the interaction models (Table 5), any PVL and AR > 2 remained significantly associated with both overall survival and cardiovascular death, whereas intra-prosthetic leak showed very large point estimates with wide confidence intervals. These very large hazard ratios should be interpreted cautiously because rare predictors in a matched sample can produce unstable estimates (sparse-data bias); however, the consistency of direction across models reinforces the clinical importance of preventing residual regurgitation and monitoring it longitudinally. The long-term adverse association of PVL (including mild PVL in some datasets) has been specifically highlighted in mid- and long-term outcome studies after TAVI [17].

Stroke was independently associated with overall mortality (HR 3.90, *p* = 0.001) in Table 6, even though it was not significant for cardiovascular death in the same model—an observation that is clinically plausible because postprocedural stroke often contributes to late disability, frailty, institutionalization, infection risk, and non-cardiac death pathways. Contemporary large-scale data demonstrate that stroke after TAVR continues to accrue over follow-up and remains a major adverse event with meaningful long-term implications [18].

Although valve size did not reach conventional statistical significance in Table 6, there was a trend for higher overall mortality with large vs. small valves (*p* = 0.057), while echocardiographic findings show that gradients were most unfavorable after TAVI in small/medium sizes. In smaller annuli, the coexistence of residual regurgitation and higher transvalvular gradients may be particularly clinically relevant and should be explicitly considered during heart team planning, including device selection, sizing strategy, and the acceptable threshold for residual leak. Reviews addressing prosthesis–patient mismatch and annulus-size-dependent hemodynamics provide a useful framework for interpreting these gradient patterns [19].

### Limitations

These findings should be interpreted within the limitations inherent to non-randomized comparative analyses. Although 1:1 propensity score matching achieved balanced baseline clinical characteristics, residual confounding cannot be fully excluded, particularly with respect to unmeasured factors such as frailty, anatomical complexity, vascular access considerations, and nuanced heart team decision making.

The extended inclusion period (2013–2024) encompasses substantial evolution in transcatheter valve technology, imaging and sizing strategies, implantation techniques, and successive iterations of the Perceval prosthesis. These temporal changes may have influenced outcomes such as paravalvular leak severity, transvalvular gradients, and conduction disturbances independently of treatment assignment. Treatment allocation was based on multidisciplinary clinical judgment and may therefore be subject to selection and referral bias not entirely captured by the available covariates.

Assessment of paravalvular and intra-prosthetic regurgitation may have been affected by operator-dependent interpretation and evolving echocardiographic techniques over time. However, all examinations were performed within a single high-volume center using standardized reporting practices. Formal inter-observer variability analysis was not performed, representing an inherent limitation of the retrospective design.

Finally, the TAVI cohort included multiple valve platforms, encompassing both self-expanding and balloon-expandable devices from different manufacturers and generations. This heterogeneity reflects real-world practice and is consistent with the study objective of comparing treatment strategies (transcatheter versus sutureless surgical AVR) rather than individual valve models. Nevertheless, device-related differences may have influenced outcomes such as paravalvular leak, conduction abnormalities, and hemodynamic performance. Subgroup analyses by valve type or generation were not performed due to limited sample sizes within individual TAVI subgroups and the risk of underpowered or misleading comparisons.

## 5. Conclusions

Overall, our findings corroborate prior reports indicating that Perceval sutureless AVR is associated with substantially lower residual regurgitation than TAVI and suggest that this advantage—together with more favorable transvalvular gradients in smaller valve sizes—may translate into improved mid-term overall survival in selected patients. In contrast, differences in permanent pacemaker implantation appear less consistent and likely reflect device-, procedural-, and center-specific factors in contemporary practice.

## Figures and Tables

**Table 1:** Sample characteristics and medical history of patients, by group, before and after propensity score matching.

	Before Propensity Score Matching					After Propensity Score Matching					
	TAVI (*n* = 424; 42.1%)		Perceval (*n* = 582; 57.9%)			TAVI (*n* = 197; 50.0%)		Perceval (*n* = 197; 50.0%)			
	*n*	%	*n*	%	SMD	*n*	%	*n*	%	SMD	*p*
Gender											
Males	198	46.7	274	47.1	0.01	89	45.2	90	45.7	0.01	>0.999 +
Females	226	53.3	308	52.9		108	54.8	107	54.3		
Chronic Pulmonary disease	107	25.2	93	16.0	0.23	45	22.8	43	21.8	0.02	0.902 +
Coronary artery disease	51	12.0	74	12.7	0.02	24	12.2	23	11.7	0.02	0.876 +
AF	35	8.3	50	8.6	0.01	16	8.1	17	8.6	0.02	0.856 +
Diabetes mellitus	168	39.6	196	33.7	0.12	74	37.6	81	41.1	0.07	0.558 +
Dialysis	10	2.4	26	4.5	0.12	4	2.0	7	3.6	0.09	0.549 +
Hypertension	330	77.8	453	77.8	0.00	152	77.2	151	76.6	0.01	>0.999 +
Dyslipidemia	309	72.9	357	61.3	0.25	131	66.5	135	68.5	0.04	0.752 +
	**Mean**	**SD**	**Mean**	**SD**		**Mean**	**SD**	**Mean**	**SD**		
Age (years)	80.86	6.60	75.27	6.83	0.83	79.77	6.21	78.82	5.32	0.09	0.106 ++
EuroSCORE II	6.10	3.97	3.38	1.63	0.90	4.53	1.93	4.44	1.88	0.05	0.450 ++
BSA	1.83	0.23	1.80	0.30	0.11	1.83	0.23	1.84	0.44	0.03	0.762 ++

SMD: Standardized Mean Difference; + McNemar test; ++ Wilcoxon signed-rank test.

**Table 2:** Immediate postoperative echocardiographic results by group.

	Procedure				
	TAVI (*n* = 197; 50.0%)		Perceval (*n* = 197; 50.0%)		
	*n*	%	*n*	%	*p* Value +
Paravalvular leaks *	46	23.4	5	2.5	<0.001
Intra-prosthetic leaks *	1	0.5	2	1.0	>0.999
Aortic regurgitation > 2	5	2.5	3	1.5	0.727

+ McNemar test; * All degrees of severity.

**Table 3:** Postoperative complications by group.

		Procedure				
		TAVI (*n* = 197; 50.0%)		Perceval (*n* = 197; 50.0%)		
		*n*	%	*n*	%	*p* Value
PPM implant	No	175	88.8	186	94.4	0.063 +
	Yes	22	11.2	11	5.6	
Pericardial effusion	No	194	98.5	193	98.0	>0.999 ++
	Yes	3	1.5	4	2.0	
AKI	No	172	87.3	177	89.8	0.428 +
	Yes	25	12.7	20	10.2	
Stroke	No	189	95.9	188	95.4	0.804 +
	Yes	8	4.1	9	4.6	
Vascular complication	None	180	91.4	-	-	
	Minor	8	4.1	-	-	
	Major	9	4.6	-	-	
		**Mean (SD)**	**Median (IQR)**	**Mean (SD)**	**Median (IQR)**	
Days until PPM implantation		31.24 (91.27)	2 (1–3)	388.8 (376.16)	190 (156–850)	0.180 ++
Overall length of stay		4.33 (3.87)	3 (2–5)	8.7 (7.2)	7 (7–9)	<0.001 ++
Length of ICU stay		1.54 (1.7)	1 (1–1.5)	2.26 (4.96)	1 (1–2)	<0.001 ++

+ McNemar test; ++ Wilcoxon signed-rank test; AKI: acute kidney injury; ICU: intensive care unit; PPM: permanent pacemaker implant.

**Table 4:** Comparison of mean pressure gradient (MPG) values between groups, within each valve size.

	Procedure				
	TAVI (*n* = 197; 50.0%)		Perceval (*n* = 197; 50.0%)		
MPG	*n*	%	*n*	%	*p* +
Small valve size	19.83 (5.68)	20 (17–22.5)	12.94 (2.18)	13 (11–14)	<0.001
Medium valve size	13.5 (4.65)	12.5 (10.5–16)	10.61 (1.61)	11 (10–12)	<0.001
Large valve size	9.77 (2.7)	9 (8–10)	9.47 (1.95)	9 (8–10)	0.872
Extra large valve size	-	-	8.45 (1.39)	8 (8–9)	-

+ Mann–Whitney test.

**Table 5:** Association of postoperative characteristics with patients’ outcome, via Cox regression.

		Main Effect		Interaction Term with Type of Procedure	
	Covariate	HR (95% CI) +	*p*	HR (95% CI) +	*p*
Overall survival	Any paravalvular leak (yes vs. no)	1.84 (1.03–3.29)	0.040	13.51 (4.29–42.54)	<0.001
	Any intra-prosthetic leak (yes vs. no)	24.98 (3.21–194.42)	0.002	7.18 (0.55–94.30)	0.133
	AR > 2 (yes vs. no)	20.06 (7.45–54.03)	<0.001	14.12 (2.34–88.94)	0.004
Cardiovascular death	Any paravalvular leak (yes vs. no)	2.62 (1.06–6.45)	0.037	11.53 (2.92–45.55)	<0.001
	Any intra-prosthetic leak (yes vs. no)	33.17 (4.19–262.57)	<0.001	3.41 (0.25–46.41)	0.357
	AR > 2 (yes vs. no)	27.26 (8.60–86.46)	<0.001	6.46 (0.95–44.03)	0.057

+ Hazard Ratio (95% Confidence Interval).

**Table 6:** Multivariate Cox regression results for overall survival and CVD death.

	Overall Survival		CVD Death	
	HR (95% CI) +	*p*	HR (95% CI) +	*p*
Age (years)	1.01 (0.97–1.04)	0.784	1.02 (0.96–1.07)	0.536
Gender (Females vs. Males)	1.02 (0.62–1.67)	0.944	1.45 (0.71–2.98)	0.306
EuroSCORE II	1.07 (0.97–1.18)	0.189	1.01 (0.85–1.19)	0.928
Procedure (Perceval vs. TAVI)	0.63 (0.35–1.12)	0.115	1.89 (0.8–4.49)	0.149
Valve size				
Medium vs. small	1.38 (0.69–2.76)	0.367	1.44 (0.56–3.73)	0.451
Large vs. small	1.98 (0.98–3.99)	0.057	1.81 (0.65–5.04)	0.258
Extra large vs. small	0.6 (0.15–2.35)	0.467	0.64 (0.11–3.59)	0.615
Paravalvular leaks (yes vs. no)	1.67 (0.9–3.1)	0.106	2.57 (0.99–6.7)	0.053
Intra-prosthetic leaks (yes vs. no)	2.06 (0.31–13.62)	0.454	1.08 (0.12–9.55)	0.947
Aortic regurgitation > 2 (yes vs. no)	19.65 (5.85–65.97)	<0.001	24 (5.8–99.23)	<0.001
PPM implant (yes vs. no)	0.49 (0.17–1.4)	0.180	0.63 (0.17–2.36)	0.489
Pericardial effusion (yes vs. no)	0.67 (0.23–1.94)	0.455	1.23 (0.32–4.69)	0.766
AKI (yes vs. no)	1.46 (0.66–3.24)	0.356	1.07 (0.26–4.37)	0.921
Stroke (yes vs. no)	3.9 (1.74–8.74)	0.001	0.88 (0.17–4.52)	0.881

+ Hazard Ratio (95% Confidence Interval).

## Data Availability

The data underlying this article are available from the corresponding author upon reasonable request.

## Supplementary Materials

The following supporting information can be downloaded at: https://www.mdpi.com/article/10.3390/medicina62030476/s1, Table S1: Pre-operative echocardiographic data by group; Table S2: Echocardiographic data at 6–12 months after surgery by group.

## Abbreviations

AR	Aortic Regurgitation
AVR	Aortic Valve Replacement
CVD	Cardiovascular Disease
ICU	Intensive Care Unit
MPG	Mean Pressure Gradient
PPI	Permanent Pacemaker Implant
TAVI	Transcatheter Aortic Valve Implantation
AKI	Acute Kidney Injury

## References

[1] Webb J.G. (2008). Percutaneous aortic valve replacement will become a common treatment for aortic valve disease. JACC Cardiovasc. Interv..

[2] Thourani V.H., Leon M.B., Makkar R., Ascione G., Szeto W.Y., Madhavan M.V., Kodali S.K., Hahn R.T., Pibarot P., Malaisrie S.C. (2025). Five-year outcomes in low-risk patients undergoing surgery in the PARTNER 3 Trial. Ann. Thorac. Surg..

[3] Forrest J.K., Yakubov S.J., Deeb G.M., Gada H., Mumtaz M.A., Ramlawi B., Bajwa T., Crouch J., Merhi W., Sang S.L.W. (2025). 5-year outcomes after transcatheter or surgical aortic valve replacement in low-risk patients with aortic stenosis. J. Am. Coll. Cardiol..

[4] Praz F., Borger M.A., Lanz J., Marin-Cuartas M., Abreu A., Adamo M., Marsan N.A., Barili F., Bonaros N., Cosyns B. (2025). 2025 ESC/EACTS Guidelines for the management of valvular heart disease. Eur Heart J..

[5] Berretta P., Andreas M., Meuris B., Langenaeken T., Solinas M., Concistrè G., Kappert U., Arzt S., Santarpino G., Nicoletti A. (2022). Sutureless and rapid deployment versus sutured aortic valve replacement: A propensity-matched comparison from the Sutureless and Rapid Deployment International Registry. Eur. J. Cardiothorac. Surg..

[6] Fischlein T., Meuris B., Folliguet T., Hakim-Meibodi K., Misfeld M., Carrel T., Zembala M., Cerutti E., Asch F.M., Haverich A. (2022). Midterm outcomes with a sutureless aortic bioprosthesis in a prospective multicenter cohort study. J. Thorac. Cardiovasc. Surg..

[7] Concistré G., Baghai M., Santarpino G., Royse A., Scherner M., Troise G., Glauber M., Solinas M. (2023). Clinical and hemodynamic outcomes of the Perceval sutureless aortic valve from a real-world registry. Interdiscip. Cardiovasc. Thorac. Surg..

[8] Biancari F., Barbanti M., Santarpino G., Deste W., Tamburino C., Gulino S., Immè S., Di Simone E., Todaro D., Pollari F. (2016). Immediate outcome after sutureless versus transcatheter aortic valve replacement. Heart Vessel..

[9] Mauri V., Gerfer S., Kuhn E., Adam M., Eghbalzadeh K., Djordjevic I., Ivanov B., Gaisendrees C., Frerker C., Schmidt T. (2021). Comparison of Self-Expanding RDV Perceval S versus TAVI ACURATE neo/TF. Thorac. Cardiovasc. Surg..

[10] Ali-Hasan-Al-Saegh S., Takemoto S., Shafiei S., Yavuz S., Rad A.A., Amanov L., Merzah A.S., Salman J., Ius F., Kaufeld T. (2024). Sutureless aortic valve replacement with Perceval bioprosthesis versus transcatheter aortic valve implantation: Systematic review and meta-analysis. J. Clin. Med..

[11] Muneretto C., Bisleri G., Moggi A., Di Bacco L., Tespili M., Repossini A., Rambaldini M. (2015). Treating the patients in the “grey-zone” with aortic valve disease: A comparison among conventional surgery, sutureless valves and transcatheter aortic valve replacement. Interact. Cardiovasc. Thorac. Surg..

[12] Desser A.S., Arentz-Hansen H., Fagerlund B.F., Harboe I., Lauvrak V. (2017). Sutureless Aortic Valve Replacement for Treatment of Severe Aortic Stenosis: A Single Technology Assessment of Perceval Sutureless Aortic Valve.

[13] Austin P.C. (2011). An introduction to propensity score methods for reducing the effects of confounding in observational studies. Multivar. Behav. Res..

[14] Meuris B., De Praetere H., Strasly M., Trabucco P., Lai J.C., Verbrugghe P., Herijgers P. (2018). A novel tissue treatment to reduce mineralization of bovine pericardial heart valves. J. Thorac. Cardiovasc. Surg..

[15] Micovic S., Nobre A., Choi J.W., Solinas M., Shehada S.-E., Torella M., Baeza C., Parrino E., Pollari F., Troise G. (2024). Early outcomes of aortic valve replacement with Perceval PLUS sutureless valve: Results of the prospective multicentric MANTRA study. J. Cardiothorac. Surg..

[16] Jilaihawi H., Makkar R. (2012). Prognostic impact of aortic regurgitation after transcatheter aortic valve implantation. EuroIntervention.

[17] Okuno T., Tomii D., Heg D., Lanz J., Praz F., Stortecky S., Reineke D., Windecker S., Pilgrim T. (2022). Five-year outcomes of mild paravalvular regurgitation after transcatheter aortic valve implantation. EuroIntervention.

[18] Okuno T., Alaour B., Heg D., Tueller D., Pilgrim T., Muller O., Noble S., Jeger R., Reuthebuch O., Toggweiler S. (2023). Long-Term Risk of Stroke After Transcatheter Aortic Valve Replacement: Insights from the SwissTAVI Registry. JACC Cardiovasc. Interv..

[19] Hahn R.T., Pibarot P. (2024). Prosthesis-patient mismatch in transcatheter and surgical aortic valve replacement. Ann. Cardiothorac. Surg..

