# Supplementary material for: Transcatheter Versus Sutureless Aortic Valve Replacement: A Propensity-Matched Single-Center Cohort Study
Source: Medicina (Kaunas). 2026 Mar 3;62(3):476. doi: 10.3390/medicina62030476 (PMC13027504; doi:10.3390/medicina62030476)
Supplement: Supplementary file 1 [file medicina-62-00476-s001.zip › medicina-4118263-supplementary.pdf]

Comparative analysis of transcatheter and sutureless aortic valve replacement: Preliminary results from a retrospective single-center cohort

Supplementary material

Table S1: Pre-operative echocardiographic data by group

	Procedure				
	TAVI (n=197; 50.0%)		Perceval (n=197; 50.0%)		
	Mean (SD)	Median (IQR)	Mean (SD)	Median (IQR)	P value++
LVEF	53.04 (8.65)	55 (50 – 60)	52.54 (7.19)	55 (50 – 55)	0.177
Aortic Valve Area	0.78 (0.17)	0.8 (0.7 – 0.9)	0.78 (0.16)	0.8 (0.7 – 0.9)	0.924
Vmax	4.3 (0.49)	4.29 (4.1 – 4.5)	4.32 (0.27)	4.3 (4.1 – 4.5)	0.761
Mean Gr	47.74 (10.56)	46 (41 – 55)	48.81 (7.7)	48 (43 – 53)	0.356

++Wilcoxon signed-rank test

LVEF: left ventricular ejection fraction

Table S2. Echocardiographic data at 6-12 months after surgery by group

		Procedure				
		TAVI (n=197; 50.0%)		Perceval (n=197; 50.0%)		
		**n**	**%**	**n**	**%**	P
Aortic regurgitation						
	None	124	63.3	192	98.0	<0.001++
	Grade I	55	28.1	1	0.5	
	Grade II	14	7.1	2	1.0	
	Grade III	2	1.0	1	0.5	
	Grade IV	1	0.5	0	0.0	
Intra-prosthetic		16	8.2	1	0.5	<0.001+
Para-prosthetic		52	26.5	3	1.5	<0.001+
		**Mean (SD)**	**Median (IQR)**	**Mean (SD)**	**Median (IQR)**	
LVEF		53.22 (7.29)	55 (50 – 60)	52.5 (8.36)	55 (50 – 60)	0.399++
mean Gr		13.77 (6.06)	12 (9 – 18)	10.33 (2.26)	10 (9 – 12)	<0.001++

+McNemar test; ++Wilcoxon signed-rank test

LVEF: left ventricular ejection fraction
